# The Role of Thoracic Medial Branch Blocks in Managing Chronic Mid and Upper Back Pain: A Randomized, Double-Blind, Active-Control Trial with a 2-Year Followup

**DOI:** 10.1155/2012/585806

**Published:** 2012-07-19

**Authors:** Laxmaiah Manchikanti, Vijay Singh, Frank J. E. Falco, Kimberly A. Cash, Vidyasagar Pampati, Bert Fellows

**Affiliations:** ^1^Pain Management Center of Paducah, 2831 Lone Oak Road, Paducah, KY 42003, USA; ^2^EMP, Anesthesiology and Perioperative Medicine, University of Louisville, Louisville, KY 40202, USA; ^3^Spine Pain Diagnostics Associates, Niagara, WI 54151, USA; ^4^Mid Atlantic Spine and Pain Physicians of Newark, DE 19702, USA; ^5^Department of Physical Medicine and Rehabilitation, School of Medical, Temple University, Philadelphia, PA 19140, USA; ^6^Psychological Services, Pain Management Center of Paducah, Paducah, KY 42003, USA

## Abstract

*Study Design*. A randomized, double-blind, active-control trial. *Objective*. To determine the clinical effectiveness of therapeutic thoracic facet joint nerve blocks with or without steroids in managing chronic mid back and upper back pain. *Summary of Background Data*. The prevalence of thoracic facet joint pain has been established as 34% to 42%. Multiple therapeutic techniques utilized in managing chronic thoracic pain of facet joint origin include medial branch blocks, radiofrequency neurotomy, and intraarticular injections. *Methods*. This randomized double-blind active controlled trial was performed in 100 patients with 50 patients in each group who received medial branch blocks with local anesthetic alone or local anesthetic and steroids. Outcome measures included the numeric rating scale (NRS), Oswestry Disability Index (ODI), opioid intake, and work status, at baseline, 3, 6, 12, 18, and 24 months. *Results*. Significant improvement with significant pain relief and functional status improvement of 50% or more were observed in 80% of the patients in Group I and 84% of the patients in Group II at 2-year followup. *Conclusions*. Therapeutic medial branch blocks of thoracic facets with or without steroids may provide a management option for chronic function-limiting thoracic pain of facet joint origin.

## 1. Introduction

Leboeuf-Yde et al. [1] showed the prevalence of thoracic pain to be 13% of the general population, in contrast to 43% with low back pain and 32% with neck pain during the past year. The data in reference to mid back or upper back pain illustrates that it is less common than chronic persistent lumbar or cervical spinal pain [1–4]. However, the degree of disability resulting from thoracic pain disorders may be similar to that of the cervical and lumbar regions [2, 4]. In interventional pain management settings, reports of thoracic pain have ranged from 3% to 23% of patients [5–9]. Even then, multiple interventional techniques performed in the thoracic spine are rising [10–20].

 Thoracic pain has been described to originate from multiple structures, including intervertebral discs and facet joints, both of which can be evaluated by proven diagnostic techniques [15, 16, 19, 21]. The accuracy of diagnostic blocks of thoracic facet joints is superior to discography. Atluri et al. [15], in a systematic review which included controlled local anesthetic blocks, showed the prevalence ranging from 34% to 48%, with false-positive rates of 42% to 58% utilizing uncontrolled blocks [6, 7, 21]. However, the treatment of thoracic pain suffers with a paucity of literature and also a lack of evidence. While there are no publications illustrating the effectiveness of intra-articular injections, and only publications with retrospective evaluations and a small number of patients who received radiofrequency neurotomy, the evidence for therapeutic medial branch blocks is emerging. There have been 3 publications evaluating the effectiveness of thoracic facet joint nerve blocks [22–24], which is much less than publications for the management of cervical and lumbar facet joint pain [19, 25, 26]. Further, these studies incorporate one observational study [22] and 2 publications of one randomized trial [23, 24]. Similarly, the study for effectiveness of pain originating from discs is also scant [27] compared to lumbar and cervical epidural injections [28–36]. In a randomized double-blind active-controlled trial, Manchikanti et al. [24] illustrated improvement in 90% of the participants, with significant pain relief and functional status improvement at 12 months.

 Significant debate surrounds the appropriate management of spinal pain with diagnostic and therapeutic modalities. The value of controlled local anesthetic blocks has been questioned and vigorously debated [34, 35, 37–42]. However, Rubinstein and van Tulder, in a systematic review [43], showed that there was strong evidence for the diagnostic accuracy of lumbar and cervical facet joint blocks in evaluating low back and neck pain based on the same studies which also evaluated thoracic pain, thus, it can be translated that the evidence for thoracic facet joint blocks is at least moderate, if not strong. The evidence for therapeutic medial branch blocks is based on only one randomized study with one-year followup. However, the mechanism of therapeutic effect of medial branch blocks in the thoracic spine is not known. Radiofrequency neurotomy has been shown to exert its effect by denaturing of the nerves. Thus, with radiofrequency neurotomy, the pain returns when the axons regenerate, requiring repetition of the radiofrequency procedure. While the mechanism of therapeutic medial branch blocks is not known, they may be repeated to reinstitute pain relief without any deleterious effects. The basis for intraarticular injections has been the inflammation of the joint; however, the effectiveness of intraarticular injections in the thoracic spine has not been evaluated.

Further arguments also surround therapeutic facet joint interventions based on a lack of understanding of placebo control and the criterion standard [44–48]. However, the criterion standard is not only limited to biopsy, but also long-term follow-up criteria [41, 42]. In fact, studies of the lumbar spine have shown the value of controlled comparative local anesthetic blocks having 80% concordant pain relief with long-term relief of up to 2 years [40–43]. Thus, even though there is ongoing debate on the diagnostic value of facet joint nerve blocks as well as therapeutic medial branch blocks, diagnostic nerve blocks appear to be an accurate method of diagnosis at the present time, with significant value as a therapeutic modality for thoracic medial branch blocks.

 This report of a double-blind randomized active controlled trial of 100 patients with 2-year results is a continuation of previous reports [23, 24]. This study was sought to evaluate the effectiveness of medial branch blocks on a long-term basis of at least a 2-year followup in patients with a confirmed diagnosis of thoracic facet joint pain by means of comparative, controlled, local anesthetic blocks based on the modified International Association of the Study of Pain (IASP) criteria of 80% pain relief, and the ability to perform previously painful movements [6, 7].

## 2. Materials and Methods

The study was performed based on Consolidated Standards of Reporting Trials (CONSORT) guidelines [49], with an approved study protocol by the Institutional Review Board (IRB), and appropriate registration with a clinical registry of NCT00355706. The study was conducted in a private practice, specialty referral center, and interventional pain management practice in the United States, utilizing the internal resources of the practice and without any external funding either from industry or from elsewhere.

### 2.1. Participants

 Study participants were recruited at the interventional pain management practice from consecutive new patients presenting with thoracic pain. One hundred patients were included and randomly assigned to one of 2 groups; either a local anesthetic only group (Group I) or a local anesthetic with steroid group (Group II), with 50 patients in each group. Patients meeting the inclusion criteria were eligible to undergo diagnostic thoracic facet joint nerve blocks. Only patients positive for controlled comparative local anesthetic blocks met the criteria for inclusion for thoracic medial branch blocks.

### 2.2. Inclusion and Exclusion Criteria

 Only patients with nonspecific mid-back or upper back pain without suspected disc herniation, radiculitis, thoracic fracture, stenosis, or intercostal neuritis were included. Further, patients suspected of disc-related pain with radicular symptoms were also excluded, based on radiologic testing and symptomatology involving radicular or chest wall pain. Patients also should have previously received conservative management with physical therapy, chiropractic manipulation exercises, drug therapy, and bed rest, and so forth, but continued to have pain.

 Further inclusion criteria were a diagnosis of thoracic facet joint pain by means of controlled comparative local anesthetic blocks; patients who were over 18 years of age; patients with a history of chronic function-limiting mid-back or upper back pain of at least 6 months duration; and patients who where competent to understand the study protocol and provide voluntary, written informed consent, and participate in the outcome measurements. A negative or false-positive response to controlled comparative local anesthetic blocks, uncontrollable to heavy opioid use (morphine equivalent of 300 mg), uncontrolled psychiatric disorders, uncontrolled medical illness, either acute or chronic, or any condition that could interfere with the interpretation of the outcome assessments, such as positioning, women who were pregnant or lactating, and patients with a history or potential for adverse reaction(s) to local anesthetics or steroids were excluded [6, 7, 24].

### 2.3. Interventions

 All patients were provided with the informed consent and protocol approved by the IRB, which described the trial's details, including side effects and the mechanism for withdrawal from the study.

#### 2.3.1. Diagnostic Thoracic Facet Joint Nerve Blocks

Controlled comparative local anesthetic blocks were employed in all patients to diagnose thoracic facet joint pain, in accordance with the modified IASP criteria, with at least 80% pain relief and ability to perform previously painful maneuvers and concordant relief lasting longer with bupivacaine than lidocaine [6, 7, 50]. The evaluation started with diagnostic medial branch blocks using 0.5 mL of 1% preservative-free lidocaine, followed by 0.5 mL of 0.25% preservative-free bupivacaine on a separate occasion, usually 3 to 4 weeks after the first injection, if positive with lidocaine. Target points were identified by the pain pattern, local or paramedian tenderness over the area of the facet joints, and reproduction of the pain with deep pressure. A positive response was considered when a patient reported at least an 80% reduction of pain assessed by the numeric rating scale (NRS) and the ability to perform previously painful movements, for at least 2 hours following the lidocaine injection, and for 3 hours or greater than the duration of relief with lidocaine when bupivacaine was used. All other responses were considered as negative.

Diagnostic medial branch blocks were performed either ipsilaterally in patients with unilateral pain or bilaterally in patients with bilateral pain. Each nerve was injected with 0.5 mL of the assigned mixture and the blocks were performed on a minimum of 2 nerves to block a single joint and 3 nerves to block 2 consecutive joints.

#### 2.3.2. Therapeutic Thoracic Facet Joint Nerve Blocks

 Following an established diagnosis, patients were enrolled in the study phase. They were then treated with therapeutic medial branch blocks under fluoroscopy in a sterile operating room with an injectate of 1 mL mixture at each level as assigned by grouping. All the blocks utilized a 22 gauge, 2-inch spinal needle. Group I patients received medial branch blocks with injection of bupivacaine 0.25%, whereas Group II participants received medial branch blocks with a mixture of bupivacaine and nonparticulate betamethasone. Nonparticulate betamethasone is a clear solution added to bupivacaine in the amount of 0.15 mg/mL.

#### 2.3.3. Additional Interventions

Patients were followed at 3-month intervals unless otherwise indicated. Thoracic facet joint nerve blocks were repeated based on the response to the prior interventions, specifically, improvement in physical and functional status. Thoracic medial branch blocks were repeated only when the reported pain levels deteriorated to below 50%, with an initial report of significant pain relief of 50% or more after the previous block. The nonresponsive patients receiving other types of treatments after stopping therapeutic lumbar facet joint nerve blocks were considered to be withdrawn from the study.

#### 2.3.4. Cointerventions

None of the patients received any specific cointerventions such as physical therapy or occupational therapy.

 However, all patients received the same co-interventions they had been receiving prior to starting the treatment, based on need, either with opioid or nonopioid analgesics and previously directed exercise program.

#### 2.3.5. Objective

 This randomized double-blind active-controlled trial was designed to determine the clinical effectiveness of therapeutic thoracic medial branch blocks of local anesthetic with or without steroids in managing chronic, disabling, thoracic pain of facet joint origin.

#### 2.3.6. Outcomes

Outcome measures included the NRS, Oswestry Disability Index (ODI), employment status, and opioid intake, with assessment at 3, 6, 12, 18, and 24 months posttreatment.

NRS represented 0 with no pain and 10 with the worst pain imaginable. The ODI was utilized for functional assessment.

 The accuracy of NRS and ODI has been widely reported [51–53].

 Significant improvement was defined as pain relief of at least 50% reduction in the NRS score, and functional status improvement illustrated by at least a 50% reduction in the ODI.

We have employed a robust outcome measure in this evaluation rather than mild decreases in pain and functional disability as described in recent evaluations [25–33, 54–57].

 Opioid intake was converted into morphine equivalence [50] based on the dose frequency and the schedule of the drug [58].

 Employment data was based on the individual's employability. Patients unemployed or employed on a part-time basis with limited or no employment due to pain were classified as employable; however, patients who chose not to work, were retired, or were homemakers who were not working but not due to pain, were considered as not employable outside.

#### 2.3.7. Sample Size

 A sample size of 50 patients for each group was determined. There were no randomized trials available to base the calculation of sample size. Previous studies of cervical and lumbar medial branch neurotomies and even epidural injections utilized a smaller number of patients [59–62]. Further, the literature evaluating the quality of individual studies has shown a sample size of 50 patients in the smallest group as acceptable [63].

#### 2.3.8. Randomization

 Of the 100 patients, 50 patients were randomly assigned into each group.

#### 2.3.9. Sequence Generation

 A computer-generated random allocation sequence generation was utilized.

#### 2.3.10. Allocation Concealment

All mixtures appeared to be identical. Patients were randomized and the solutions were prepared appropriately by the operating room nurse assisting with the procedure.

#### 2.3.11. Implementation

After the patients had met the inclusion criteria, one of the 3 nurses assigned as coordinators of the study enrolled and assigned them to their respective groups. All patients were invited to enroll in the study if they met inclusion criteria.

#### 2.3.12. Blinding

The random allocation was not revealed to either the participants or the physician. In addition, all the study patients were mixed with other patients with no specific indication that patients were participating in the study.

Patients were unblinded early if they requested to be unblinded or after completing 24 months of the study. Patients were provided with an opportunity to discontinue or withdraw from the study for lack of pain relief or for any other reason. All patients with loss of followup were considered to be withdrawn.

#### 2.3.13. Statistical Methods

Chi-squared statistic, Fisher's exact test, paired *t*-test, and one-way analysis of variance were used to analyze the data.

Chi-squared statistic was used to test the differences in proportions. Fisher's exact test was used wherever the expected value was less than 5; a paired *t*-test was used to compare the pre- and posttreatment results of average pain scores, the ODI measurements and combined NRS and ODI scores at baseline versus 3, 6, 12, 18, and 24 months. The *t*-test was performed for comparison of mean scores between groups. One-way analysis of variance was used for comparison of means among groups.

#### 2.3.14. Intent-to-Treat-Analysis

An intent-to-treat-analysis was performed on all patients utilizing the last followup data, with application of initial data in the patients who dropped out of the study without further follow-up after the first treatment. Sensitivity analysis was performed utilizing best case, worst case, and last follow-up scores scenarios.

## 3. Results

### 3.1. Participant Flow

**Figure 1:**
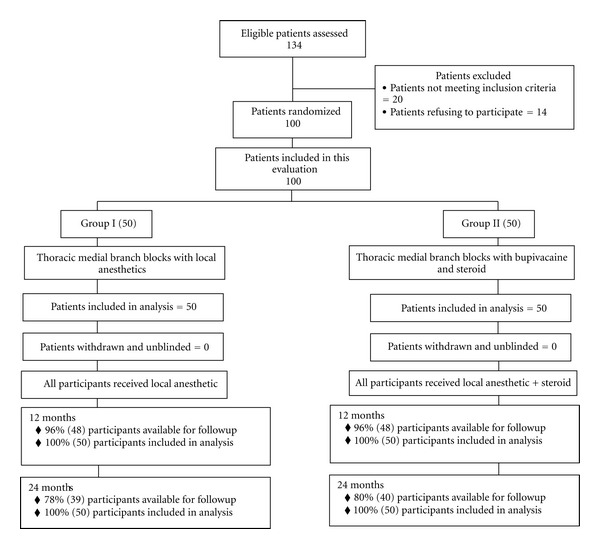
Schematic presentation of participant flow at 2-year followup.

Participant flow is illustrated in Figure 1.

### 3.2. Recruitment

The recruitment period lasted from April 2003 through August 2009.

### 3.3. Baseline Data

Demographic characteristics are illustrated in Table 1.

The number of joints was as follows: 3 joints were involved in 23% of the participants, 4 joints were involved in 28% of the participants, 5 joints were involved in 33% of the participants, and 6 joints were involved in 16% of the participants. Bilateral involvement was seen in 68% of the participants.

### 3.4. Pain Relief and Functional Assessment

Table 2 presents the results of repeated measures analysis. There were no significant differences between groups with regards to average pain scores and Oswestry Disability Index. However, there were significant differences within groups by time (*P* = 0.0000) for to average pain scores and Oswestry Disability Index.

**Figure 2:**
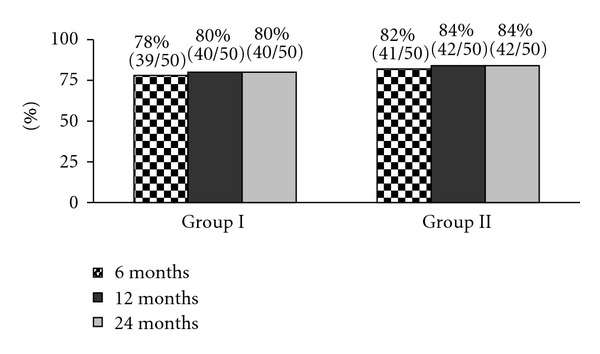
Proportion of patients with significant reduction in numeric rating score and Oswestry Disability Index (≥50% reduction from baseline).

A post hoc analysis indicates that all the mean differences between baseline and with other time point's scores were significant at the 0.05 level.  Figure 2 illustrates the proportion of patients with significant pain relief and reduction in at least 50% of the disability scores from baseline.

### 3.5. Procedural Characteristics

Table 3 illustrates the therapeutic procedural characteristics with average weeks of pain relief per procedure over a period of 2 years.

### 3.6. Employment Characteristics

 Employment characteristics are illustrated in Table 4.

### 3.7. Opioid Intake

Table 5 presents the results of repeated measures analysis for opioid intake. There were no significant differences in opioid intake within group by time.

### 3.8. Adverse Events

There were no serious adverse events reported in the study including infection, pneumothorax, nerve root trauma, or spinal cord trauma.

## 4. Discussion

The first randomized, double-blind, active-controlled trial of 100 patients with chronic function-limiting thoracic pain of facet joint origin, using therapeutic thoracic medial branch blocks, showed significant improvement with pain relief and functional status improvement in 80% of the patients in Group I and 84% in Group II at 2-year followup. This study also showed an average number of procedures of 6 over a period of 2 years. Patients experienced 84.7 ± 26.1 weeks of relief in Group I and 88.7 ± 22.1 weeks of relief in Group II. The study illustrated the average relief per procedure as 20.4 ± 20.8 weeks in Group I and 17.4 ± 14.4 in Group II with steroids with relief per procedure from 2 weeks to 2 years. While there was no significant difference in opioid intake or employment characteristics, employment characteristics showed that all the eligible participants were employed at the end of one year and 2 years with one fewer participant in Group I because of retirement. Thus, pain relief and improvement in functional status were significant. Strict criteria were utilized for diagnosing facet joint pain with controlled comparative local anesthetic blocks to avoid the criticism of including patients without facet joint pain in the study.

 The results of the current study are similar to a previously reported observational study [22] and 2 preliminary reports of thoracic facet joint pain [23, 24]. This is the first randomized double-blind controlled trial evaluating the effectiveness of thoracic medial branch blocks in managing chronic persistent function-limiting mid back and upper back pain of thoracic facet joint origin. This study also is similar to the published results of randomized trials of cervical and lumbar facet joint nerve blocks [25, 26].

 Criticisms may be included in reference to placebo control. While it is admitted that the lack of placebo control is a drawback, placebo is difficult in any type of neural blockade, apart from the ethical issues, with any interventional techniques. So-called placebo controlled studies have been associated with design flaws, because they lack an understanding of true placebo and do no consider the nocebo effect [35, 36, 40, 61, 64–68]. The only properly conducted placebo-controlled trial with transforaminal epidural injection showed sodium chloride solution, when injected into an inactive structure, has no effect [69]. The effect of any solution injected into a closed space, such as an intraarticular space, or epidural space, or over a nerve, has not been appropriately evaluated. In fact, multiple studies have illustrated a significant effect for sodium chloride solution, either injected into the epidural space, intraarticularly, over the nerves, and so forth [61, 66–68]. Further, a multitude of differences have been published with injection of either sodium chloride solution or dextrose, both considered as placebo [70–73]. In addition, the argument that local anesthetics are placebo is not tenable [35, 36, 44, 45, 47, 48]. Finally, the evidence in this paper leads to the conclusion that the effect of local anesthetic on thoracic medial branch blocks cannot be attributed to the placebo effect, as long-lasting relief of 2 years with multiple interventions in a substantial proportion of patients is impossible to obtain with a placebo effect. However, the limitations of lack of placebo must not be underestimated. If feasible, a placebo-controlled study with appropriate design that includes not injecting the placebo solution over the medial branches, such as the one designed by Ghahreman et al. [69] and publication of subsequent results over a long period of time of 2 years would be valid and provide conclusive knowledge on the issue of placebo-control blocks.

 In the era of comparative effectiveness research (CER) and evidence-based medicine (EBM) and escalating health care costs, active controlled trials are important for providing practice patterns [64, 65, 74–77]. This study was conducted in a practical setting, repeating the procedures only with return of pain and deterioration in functional status. This is the first and the largest study with the longest followup regarding an interventional technique for managing thoracic facet joint pain. This study, similar to other studies, may resolve the issue of adding steroids to local anesthetic and therapeutic medial branch blocks. The evidence once again illustrates that there is no significant role for steroids in thoracic medial branch blocks. Even though the relief is limited over a period of 2 years, as long as 20 weeks on average with each procedure, long-term relief can be achieved by prudent management of the patients with appropriate selection. However, the issue of local anesthetics providing long-acting relief may still be questioned. The basis in general for intraarticular injections has been that there is inflammation, and steroids are used to treat the inflammation. The literature is replete with descriptions of epidural corticosteroids providing a certain level of efficacy by their anti-inflammatory, immunosuppressive, antiedema effect, and inhibition of neural transmission within the C fibers [78–81]. However, local anesthetics also have been described to provide long-term symptomatic relief, even though the mechanism of this relief is described as an enigma and rather widely debated [22–33, 54–57, 74, 75, 81–85]. Local anesthetics have been postulated to function by suppression of nociceptive discharge [86], blockade of the axonal transport [82, 83], blockade of the sympathetic reflex arc and *sensitization* [84, 87], and by exerting anti-inflammatory effects [85]. Further, the lack of superiority of added steroids has been illustrated not only in clinical studies [22–33, 54–57], but also in experimental studies [88, 89].

In summary, the results of this study present a real-world example describing patients in a private interventional pain management practice setting, with appropriate selection and judicious use of modalities, with results generalizable to similar settings. However, caution must be exercised in applying these results to the general population unless the same methodology is used for both diagnosis and therapy. The generalizability of the findings of this study might only be feasible if studies are published using large populations in multiple settings.

## 5. Conclusion

This randomized, double-blind, active-controlled trial report demonstrates that thoracic facet joint pain diagnosed by controlled comparative local anesthetic blocks may be treated with thoracic medial branch blocks of local anesthetics with or without steroids with similar results.

## Figures and Tables

**Table 1:** Baseline demographic characteristics.

		Group I (*N* = 50)	Group II (*N* = 50)	*P* value
Sex	Male	38% (19)	36% (18)	0.836
	Female	62% (31)	64% (32)	
Age	Mean ± SD	44.7 ± 11.7	42.8 ± 12.3	0.431
Height (inches)	Mean ± SD	67.5 ± 3.9	65.9 ± 3.9	0.042
Weight (lbs.)	Mean ± SD	197.6 ± 53.2	172.3 ± 37.1	0.007
BMI		30.2 ± 6.6	28.0 ± 3.3	0.079
Duration of pain (months)	Mean ± SD	78.0 ± 68.8	77.0 ± 73.6	0.994
Mode of onset of Pain	Nontraumatic	68% (34)	72% (36)	0.663
	Traumatic	32% (16)	28% (14)	
History of previous thoracic surgery		2% (1)	6% (3)	0.617

Group I = bupivacaine only.

Group II = bupivacaine and steroid.

**Table 2:** Comparison of numeric rating scale for pain and Oswestry Disability Index score summaries at four time points.

Time points	Numeric pain rating scale		Oswestry disability index	
	Mean ± SD		Mean ± SD	
	Group I (*N* = 50)	Group II (*N* = 50)	Group I (*N* = 50)	Group II (*N* = 50)
Baseline	7.9 ± 0.9	7.8 ± 1.0	27.1 ± 6.6	27.5 ± 5.8
3 months	3.1* ± 0.9(94%)	3.1* ± 0.7(96%)	13.0* ± 4.9(80%)	11.6* ± 3.7(88%)
6 months	3.0* ± 0.9(94%)	3.2* ± 0.8(94%)	13.0* ± 4.2(78%)	11.9* ± 3.8(82%)
12 months	3.2* ± 0.9(90%)	3.1* ± 1.0(90%)	12.0* ± 4.0(80%)	11.8* ± 3.9(84%)
18 months	3.0* ± 1.0(88%)	3.1* ± 0.9(90%)	12.1* ± 4.9(80%)	11.7* ± 3.9(82%)
24 months	3.1* ± 1.2(86%)	3.1* ± 1.0(88%)	11.7* ± 4.9(82%)	11.0* ± 4.2(86%)
Group difference	0.964		0.560	
Time difference	0.000		0.000	
Group by time interaction	0.884		0.112	

*Significant difference with baseline values within the group (*P* < 0.05).

() Illustrates proportion with significant pain relief (≥50%) from baseline.

Group I = bupivacaine only.

Group II = bupivacaine and steroid.

**Table 3:** Therapeutic procedural characteristics with procedural frequency, average relief per procedure, and average total relief in weeks over a period of 2 years.

	Group I (50)	Group II (50)	Combined (100)
One year			
Average number of procedures per one year (range)	3.5 ± 1.0(1–5)	3.5 ± 0.9(1–4)	3.5 ± 0.9(1–5)
Average total relief per year (weeks) (range)	47.2 ± 10.1(4–52)	46.3 ± 8.4(16–52)	47.0 ± 9.0(4–52)
Average relief per procedure (range)	15.8 ± 10.5(2–52)	13.6 ± 3.6(10–26)	15.1 ± 8.6(2–52)
Two years			
Average number of procedures per two years (range)	5.6 ± 2.4(1–8)	6.2 ± 2.2(1–8)	5.9 ± 2.3(1–8)
Average total relief per two years (weeks) (range)	84.7 ± 26.1(4–104)	88.7 ± 22.1(26–104)	86.7 ± 24.1(4–104)
Average relief per procedure (range)	20.4 ± 20.8(2–104)	17.4 ± 14.4(8–104)	18.9 ± 17.8(2–104)

**Table 4:** Employment characteristics.

Employment status	Group I			Group II		
	Baseline	12 months	24 months	Baseline	12 months	24 months
Employed part-time	5	5	2	1	3	3
Employed full-time	10	14	16	14	16	16
Unemployed	4	0	0	3	0	0
Total employed	15	19	18	15	19	19

Eligible for employment	19	19	18	18	19	19

Housewife	2	2	1	3	3	3
Disabled	23	23	24	27	25	26
Over 65 years of age	6	6	7	2	3	2

Total number of patients	50	50	50	50	50	50

**Table 5:** Opioid intake (morphine equivalence mg).

Narcotic intake	Group I (50)	Group II (50)
(Morphine equivalence mg)	Mean ± SD	Mean ± SD
Baseline	48.0 ± 53.75	47.9 ± 48.6
3 months	38.0 ± 44.2	40.3 ± 33.9
6 months	38.2 ± 46.1	39.3 ± 34.8
12 months	37.6 ± 38.4	37.8 ± 33.2
18 months	37.6 ± 46.4	38.7 ± 35.2
24 months	37.6 ± 46.4	38.7 ± 35.2
Group difference	0.899	
Time difference	0.108	
Group by time interaction	0.999	
